# A conversation with Aaron Richterman, MD, MPH, Assistant Professor of Medicine (Infectious Diseases), Raymond and Ruth Perelman School of Medicine at the University of Pennsylvania

**DOI:** 10.1017/cts.2025.25

**Published:** 2025-02-28

**Authors:** Clinical Research Forum

**Affiliations:** Clinical Research Forum, Washington, DC, USA

This article is part of a series of interviews with recipients of Clinical Research Forum’s Top 10 Clinical Research Achievement Awards. This interview is with Aaron Richterman, MD, MPH, Assistant Professor of Medicine (Infectious Diseases), Raymond and Ruth Perelman School of Medicine at the University of Pennsylvania. Dr Richterman is a physician-scientist whose research focuses on the relationship between poverty, food security, and individual and population outcomes for infectious diseases, as well as the use of the social safety nets to improve these outcomes. He received a 2024 Top 10 Clinical Research Achievement Award for “The effects of government-led cash transfers on all-cause mortality in low- and middle-income countries.” *The interview has been edited for length and clarity.*

## When did you first become interested in clinical research?

I was a math major in college and shortly after I graduated, I worked at the National Institutes of Health in a translational immunology lab focused on HIV. That experience gave me a rich lens to think about a lot of different things, not only the biology of the HIV virus, which is incredible, but also the immune response to it, treatments, prevention, and a whole range of social, cultural, and political forces that have let this pandemic play out the way that it has over the last 40 years. I found all of that really compelling and it’s what got me interested in pursuing medicine. Then, in medical school, even though I tried and liked different specialties and subject areas, I kept getting drawn back to infectious disease, HIV, and the many different angles you need to think about to effectively care for somebody with HIV.

## Did your interest in HIV lead to your MPH?

Yes. I did my residency in internal medicine at Brigham and Women’s Hospital and while there, I earned my MPH. As part of that program, I was able to work on a project related to global health, and I spent most of a year in rural Haiti doing ambulatory HIV and tuberculosis care.

## What did you learn from your experience in Haiti?

Treatment for HIV is now fairly straightforward and for most people, it involves taking a pill once a day. These drugs are much better than they were in terms of side effects and toxicities, and they’re available for free in most places. However, when I was working in Haiti I saw 15–20 outpatients a day, and what kept coming up again and again was that people couldn’t take their medicine because they didn’t have enough food to eat. This was the case for about half of the people I saw. It was really striking, and it got me interested in how food insecurity influences health outcomes.

## So these patients couldn’t benefit from HIV treatment advances because of food insecurity?

Right. HIV is a good lens to think about because even though we now have incredible treatments and prevention, there still are raging HIV epidemics with many people getting infected and many people dying each year. In other words, the benefit from the biomedical advances can level off at a certain point because we’re not addressing the upstream factors. This can be true for other diseases, as well. While I was in Haiti, I also did some work looking at the relationship between food insecurity and cholera. Cholera has long been known to be associated with contaminated water. However, our research showed that people who are food insecure also have a much higher risk of being exposed to the bacteria that causes cholera.

## How did your interests shift from food insecurity to poverty reduction?

When I came to the University of Pennsylvania in 2020, I was interested in moving my research from the observational realm – characterizing what food insecurity affects and the ways it does that – to the more actionable realm – what we can do about it and how can we effectively improve health outcomes. What we’ve seen over time is that for many diseases, the first thing that happens is that social supports are put in place, and people benefit from them. Then, once biomedical advances occur, the social supports start to disappear because more and more of the available funds are spent on the medicine. I started to think about how these things shouldn’t be so siloed and how it could be plausible that the health impacts of concentrated investment in anti-poverty could be very large. There is limited evidence to support that idea, so I wanted to get a better understanding of the big-picture consequences of anti-poverty efforts like cash transfer programs.

## Why are anti-poverty efforts siloed from health-related programs?

Traditionally, people think about health programs and anti-poverty programs as completely separate. For example, in the U.S., we have a big food security program called SNAP (Supplemental Nutrition Assistance Program). It’s not considered a health program, but it has health effects. When we think about the costs and benefits of a program like SNAP, it would be useful to look at the benefits across all sectors, but that usually doesn’t happen.

## What did the award-winning research find?

Our research looked at the effects of government-led anti-poverty cash transfer programs on population-level mortality rates. We included programs from 37 countries impacting about 7 million people, and we found that the cash transfer programs led to a 20% reduction in mortality rates among adult women and an 8% reduction in mortality rates among children under the age of five. In addition, we found a sort of dose response, where the programs that had the highest coverage rates or had the greatest transfer amounts tended to have the largest effects on mortality. We also found that there were the largest effects on mortality in countries with the lowest life expectancies and the poorest healthcare infrastructure.

## Why are these findings important?

A lot of countries have rolled out programs that give people cash through some kind of targeting mechanism, usually to poor families, but it could be to older people, children, or people with a disability. These programs have been out long enough for us to start identifying the population health effects, although most of the research that has been done is smaller scale, for individual programs. Our study gives a wider view. We showed that when there is a large program, there can be general health benefits beyond the people who receive the cash.

## Is there a way to assess the cost-effectiveness of these programs?

We haven’t done a formal cost-effectiveness analysis, but if we use some basic assumptions, these cash transfer programs do seem to be a particularly cost-effective way to invest in population health. For instance, if you look across the whole population we studied, the cost per life saved overall is about $11,000. Over time, the benefits can get bigger while the upfront costs of implementing a program decrease. That means when you look out after five years, the cost per life saved gets closer to $5,000, which is a benchmark established by GiveWell, a nonprofit dedicated to finding outstanding giving opportunities. Cash transfers redistribute purchasing power to poor families, who go on to spend it on many other things, benefiting the local economy. It’s not comparable to malaria nets or drugs, which are end products and don’t generate this additional economic activity.

## How does your work as a physician impact your research?

I’m a physician scientist and what I bring to the table is the perspective I gain from having a clinic every week. I hear directly from people in a very vulnerable, privileged way, about what’s going on in their lives. Getting that perspective is critical.

## Why is getting the patient’s perspective critical?

These individualized perspectives give me insight. They are a tremendous source of hypotheses, and I’m able to combine them with the bigger picture. I often work with researchers who do not see patients, and I can bring the understanding that comes from talking with and hearing from patients in a non-directed way about what is driving the issues we are researching. Working in the clinic, I hear about what matters to people. Then, I can turn to the research and think about those issues in a more rigorous population-based way. That’s what makes this career great. It’s really rewarding to be able to do that.

